# Transdiagnostic, Connectome-Based Prediction of Memory Constructs Across Psychiatric Disorders

**DOI:** 10.1093/cercor/bhaa371

**Published:** 2020-12-21

**Authors:** Daniel S Barron, Siyuan Gao, Javid Dadashkarimi, Abigail S Greene, Marisa N Spann, Stephanie Noble, Evelyn M R Lake, John H Krystal, R Todd Constable, Dustin Scheinost

**Affiliations:** Department of Psychiatry, Yale School of Medicine, New Haven, CT 06510, USA; Department of Anesthesiology and Pain Medicine, University of Washington, Seattle, WA 98112, USA; Department of Biomedical Engineering, Yale School of Engineering and Applied Science, New Haven, CT 06520, USA; Department of Computer Science, Yale University, New Haven, CT 06520, USA; Interdepartmental Neuroscience Program, Yale University, New Haven, CT 06520, USA; Irving Medical Center, Columbia University, New York, NY 10032, USA; Department of Radiology and Biomedical Imaging, Yale School of Medicine, New Haven, CT 06520, USA; Department of Neurosurgery, Yale School of Medicine, New Haven, CT 06520, USA; Department of Psychiatry, Yale School of Medicine, New Haven, CT 06510, USA; Department of Radiology and Biomedical Imaging, Yale School of Medicine, New Haven, CT 06520, USA; Department of Neurosurgery, Yale School of Medicine, New Haven, CT 06520, USA; Department of Biomedical Engineering, Yale School of Engineering and Applied Science, New Haven, CT 06520, USA; Department of Radiology and Biomedical Imaging, Yale School of Medicine, New Haven, CT 06520, USA; Department of Statistics and Data Science, Yale University, New Haven, CT 06520, USA; Child Study Center, Yale School of Medicine, New Haven, CT 06520, USA

**Keywords:** (<5): prediction, functional connectivity, machine learning, psychiatry, transdiagnostic

## Abstract

Memory deficits are observed in a range of psychiatric disorders, but it is unclear whether memory deficits arise from a shared brain correlate across disorders or from various dysfunctions unique to each disorder. Connectome-based predictive modeling is a computational method that captures individual differences in functional connectomes associated with behavioral phenotypes such as memory. We used publicly available task-based functional MRI data from patients with schizophrenia (*n* = 33), bipolar disorder (*n* = 34), attention deficit hyper-activity disorder (*n* = 32), and healthy controls (*n* = 73) to model the macroscale brain networks associated with working, short- and long-term memory. First, we use 10-fold and leave-group-out analyses to demonstrate that the same macroscale brain networks subserve memory across diagnostic groups and that individual differences in memory performance are related to individual differences within networks distributed throughout the brain, including the subcortex, default mode network, limbic network, and cerebellum. Next, we show that diagnostic groups are associated with significant differences in whole-brain functional connectivity that are distinct from the predictive models of memory. Finally, we show that models trained on the transdiagnostic sample generalize to novel, healthy participants (*n* = 515) from the Human Connectome Project. These results suggest that despite significant differences in whole-brain patterns of functional connectivity between diagnostic groups, the core macroscale brain networks that subserve memory are shared.

## Introduction

Memory is a fundamental construct in cognitive and clinical neuroscience. Because the ability to remember and manipulate information is critical to everyday life, attempts to understand individual differences in memory have led to an increasingly nuanced breakdown of different memory constructs, each attempting to capture and explain variability in memory function across people (Baddeley 1988). For example, working memory refers to a task-limited storage and manipulation of information (Christophel et al. 2017). In contrast, short- and long-term memory are the capacity for holding, but not manipulating, information that is readily available for a short or long period of time (Norris 2017).

Deficits in specialized forms of memory are thought to be characteristic of different mental illnesses. Working memory deficits, for example, have long been observed in patients with schizophrenia (SCZ), and functional magnetic resonance imaging (fMRI) studies have indicated that increased activity in the dorsolateral prefrontal cortex at higher working memory loads may provide a brain correlate of this deficit (Callicott et al. 2003). While similar memory deficits are observed in a range of psychiatric disorders (Etkin et al. 2013), it is unclear whether these deficits arise from a shared neurobiological dysfunction across disorders or from ones that are unique to each disorder. Finally, if shared correlates across psychopathology exist, it is also unclear whether these correlates can be observed in healthy individuals, and whether individual differences in memory function arise from these macroscale brain networks.

Several emerging works support the goal of elucidating transdiagnostic, brain-phenotype associations. First, group-level brain imaging studies have shown that the same brain networks (Goodkind et al. 2015; Vanasse et al. 2018) are implicated in a range of psychiatric diseases. Second, characterizing mental health disorders by disrupted cognitive processes with a distinct neurobiological cause, which may transcend diagnostic group, promises greater validity than traditional approaches (Insel et al. 2010; Insel and Sahakian 2012). Third, in the context of mental health, “health” and “disease” are increasingly viewed not as strict, binary groupings, but rather as fuzzy boundaries along a continuous spectrum that may shift depending on how you define health and disease (Holmes and Yeo 2015). Finally, a wide range of brain imaging, behavioral, and genomic studies have shown pervasive heterogeneity within diagnostic group and overlapping distributions of cognition, including measures of memory, across diagnostic group and healthy controls (Consortium C-DGotPG 2013; Holmes and Patrick 2018).

Given growing evidence that complex cognitive processes are represented by distributed, rather than focal, patterns of activity, functional connectivity is particularly well suited to elucidate distributed, transdiagnostic correlates of memory. Functional connectomes, or functional connectivity matrices, represent how brain activity from spatially distinct regions covaries over time. Each brain region is considered a node, and temporal correlation in the activity between two nodes are considered functional connections or edges. An individual’s connectome has been shown to be unique to an individual (Finn et al. 2015), stable over a period of years (Horien et al. 2019), and predictive of clinical and cognitive traits in novel subjects (Rosenberg et al. 2016; Dubois et al. 2018; Lake et al. 2019). Importantly, predictive models of memory from connectomes have shown promise in understanding the networks underlying working memory impairment (Yamashita et al. 2018; Avery et al. 2020). Functional connectivity derived from task-based data may be particularly well suited for investigating transdiagnostic properties of memory. While connectomes are typically generated using resting-state fMRI data, using task-based data has been shown to improve the prediction of individual cognitive traits and more clearly delineate brain–behavior associations (Greene et al. 2018; Jiang et al. 2020).

We sought to uncover models using task-based connectomes that predict memory function, to test if these models generalize across diagnostic groups, and to test whether whole-brain patterns of functional connectivity differed across psychiatric patients and healthy controls. To this end, we used connectome-based predictive modeling (CPM) (Shen et al. 2017) to train and test a model for working, short-, and long-term memory measures across patients diagnosed with SCZ, bipolar affective disorder (BPAD), and attention deficit hyper-activity disorder (ADHD), and healthy individuals. In line with our previous work, we show that individual differences in memory function are related to individual differences within shared networks across diagnosis, as demonstrated by the successful use of a single model to predict memory scores in all groups. Next, we show that—in addition, to these shared networks of memory—whole-brain patterns of functional connectivity differ across diagnostic groups, using a mass multivariate analysis (MMA) (Dadashkarimi et al. 2019). While these differences were overrepresented in similar canonical brain networks as the memory models, the specific node and network patterns of these differences were distinct. Finally, we test whether the model trained on patient data generalizes to novel subjects from an independent, external dataset of healthy individuals. Altogether, our results suggest that despite significant differences in whole-brain patterns of functional connectivity between diagnostic groups, the macroscale brain networks that subserve memory are shared across these groups.

## Materials and Methods

We used two independent datasets in our analyses. We used the UCLA Consortium for Neuropsychiatric Phenomics (CNP) for the primary analysis and the Human Connectome Project (HCP) 900 Subjects release—the most recent release available when this work began—for external validation.

### CNP Participants

**Figure 1:**
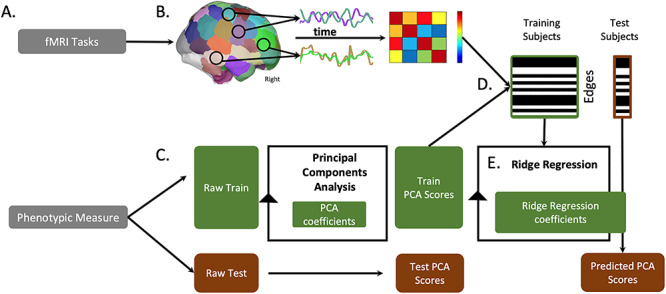
Overview of processing pipeline. (*A*) We use six fMRI tasks and five categories of phenotypic measures from the Neuropsychiatric Phenomics Consortium dataset (see Methods and Supplementary Materials). (*B*) We preprocess and divide fMRI volumes using the Shen 268 node atlas. We then create a cross-correlation matrix of internode connectivity, hereafter described as edges. (*C*) We separate behavior and (*D*) fMRI data into train and test groups. We perform a principal component analysis to summarize one behavioral construct score per subject; we use the training data’s PCA coefficients to transform the behavioral test data into component space. (*D*) Across training subjects, we correlate each edge to the phenotypic scores and restrict subsequent analyses to edges with a correlation strength above *P* < 0.01 (see Supplementary Materials for different statistical thresholds). (*E*) We use a ridge regression algorithm to train a predictive model wherein edges from all 6 fMRI tasks predict a phenotypic score. We apply this model to the selected edges to predict phenotypic scores for each individual in the test group. Model performance measures are described in Methods.

The overarching goal of the UCLA CNP is to understand the dimensional structure of memory and cognitive control in patients and healthy controls. To this end, the CNP includes extensive set of MRI and behavioral data which is openly available through the Open fMRI project, and is formatted according to the Brain Imaging Data Structure (BIDS) standard. Details of the CNP data may be referenced elsewhere (Poldrack et al. 2016). From the CNP data, we restricted this larger sample to subjects who had full-brain structural images and task-based functional MRI acquisitions during the balloon analog risk task (BART), Paired Associative Memory encoding (PAM-E), Paired Associative Memory retrieval (PAM-R), Spatial Working Memory Capacity (SCAP), Stop Signal (SS), and Task Switching (TS) (see Fig. 1*A*). We excluded 100 subjects (55 controls, 20 SCZ, 14 bipolar, and 11 ADHD) because the above whole-brain image volumes were unavailable for all 6 tasks, because they had excessive head motion defined a priori as >0.15 mm grand mean frame-to-frame displacement across all 6 tasks, or >0.20 mm mean frame-to-frame displacement on any individual task. After these criteria, we included 172 subjects (HC = 75, SCZ = 30, BPAD = 35, and ADHD = 32). From the UCLA neuropsychological battery, working memory was measured using the Wechsler Memory Scale (WMS) symbol span, WMS digit span, and Wechsler Adult Intelligence Scale (WAIS) letter–number sequencing; short-term memory was measured using the Verbal recall I, and California Verbal Learning Task (CVLT) short-delay free recall; and long-term memory was measured using Verbal recall II, CVLT long-delay free recall, and CVLT scene recognition overall accuracy.

### CNP Connectivity Processing

Whole-brain functional connectivity was assessed as described previously (Finn et al. 2017; Greene et al. 2018). Standard preprocessing procedures were applied including brain extraction, motion correction, nonlinear registration to MNI-152 standard space, and spatial smoothing (6-mm FWHM). Next, the task fMRI data was further processed by removal of motion-related components of the signal; regression of mean time courses in white matter, cerebrospinal fluid, and gray matter; removal of the linear trend; and low-pass filtering. Task-based connectomes were calculated using the “raw” task time courses, without the removal of task-evoked activity. Using the Shen 268 node atlas, for every node, a mean time course was calculated by averaging across voxels within node (see Fig. 1*B*). We selected the Shen 268 node atlas in line with previous work showing that Pairwise Pearson correlation was computed between all pairs of nodes. Correlations were Fisher *z*-transformed to yield symmetric 268 × 268 connectivity matrices.

### HCP Participants

HCP S900 Release Data were obtained from the HCP 900-participant release of December 2015 (Essen et al. 2013). From this dataset, we restricted our analyses to those individuals who participated in all the nine fMRI conditions (7 task, 2 rest), whose mean frame-to-frame displacement was less than 0.1 mm and whose maximum frame-to-frame displacement was less than 0.15 mm, and for whom memory measures were available (*n* = 515; 241 males; ages 22–37). For measures of memory, we selected the list sorting working memory and the picture sequence memory task from the NIH Toolbox for Assessment of Neurological and Behavioral function (http://www.nihtoolbox.org) and Penn word memory test from the University of Pennsylvania Computerized Neurocognitive Battery. These tasks have previously been shown to cluster into a single memory factor (Dubois et al. 2018).

### HCP Connectivity Processing

The HCP minimal preprocessing pipeline was used on these data (Glasser et al. 2013), which includes artifact removal, motion correction, and registration to standard space. All subsequent preprocessing was performed in BioImage Suite and was identical to the CNP processing.

### Predictive Modeling Framework

Figure 1 shows an overview of our predictive modeling approach. Given that a single behavioral measurement is an incomplete approximation of a behavioral construct with substantial noise due to subject and administration variability, we concatenated across multiple individual measures (i.e., working, short-, and long-term memory). We used a principal components analysis (PCA) to create latent memory phenotypes for predictive modeling with CPM. A similar strategy has been successfully employed to create a phenotypic measure of intelligence across individual measures of crystallized ability, processing speed, visuospatial ability, and memory (Dubois et al. 2018). To maintain separate train and test groups, for each iteration, each PCA was limited to the training datasets and the PCA coefficients applied to the test dataset (see Fig. 1*C*).

To generate predictive models of latent memory phenotypes, we combined all task connectomes into a single predictive model using a modified CPM framework based on ridge regression, called ridge regression CPM (rCPM; Gao et al. 2010). Using multiple connectomes improves predictive modeling and facilitates a more holistic characterization of brain–behavior associations (Gao et al. 2019; Jiang et al. 2020). However, there is a high degree of similarity between connectomes from different tasks, and edges from these connectomes are not independent. In brief, rCPM accounts for these dependencies in a principled manner. For feature selection, we use a significance threshold of *P* < 0.01 to select edges that are positively and negatively correlated with the latent memory phenotype across individuals in the training data. We controlled for motion at this feature selection step using partial correlation (Hsu et al. 2018). A schematic of additional analysis to control for confounds such as motion, *P*-value, and edge number can be found in Supplementary Figure 1.

### Model Validation

We used three validation approaches, each with an explicit goal. First, we used 10-fold cross-validation to train transdiagnostic models of memory. For 10-fold cross-validation, we randomly divided the whole sample (*N* = 172)—regardless of diagnostic group—into 10, approximately equal-sized groups; on each fold, the model was trained on 9 groups and tested on the excluded 10th group. Unless otherwise specified (cf., the Supplementary Materials), we repeat this procedure for 1000 random divisions. Second, we used leave-one-group-out cross-validation to test whether a model trained on all but a single diagnostic category generalizes to that left-out group (see Fig. 3). We did not iterate over the leave-one-group-out analysis since the possibilities are exhausted with one set. Third, we used an external validation set to test whether models trained on UCLA data generalize to the independently acquired HCP dataset.

### Assessing Prediction Performance

For the 10-fold cross-validation analyses, we evaluated model performance with a cross-validated *R^2^*
 

$$ {R}_{CV}^2=1-\frac{\sum_{i=1}^n{\left({y}_i-\hat{y}\right)}^2}{\sum_{i=1}^n{\left({y}_i-\overline{y}\right)}^2} $$.

$\sqrt{R_{CV}^2}$ is reported as it is comparable to, but less biased than, the typically used Pearson correlation value when using cross-validation. In the text, we report the median $\sqrt{R_{CV}^2}\ \mathrm{value}$ for 1000 random 10-fold divisions. To assess significance of $\sqrt{R_{CV}^2}$, we use permutation testing, where we randomly shuffle the correspondence between behavioral variables and connectivity matrices 1000 times and rerun the rCPM analysis with the shuffled data to generate null distributions of $\sqrt{R_{CV}^2}$. Based on these null distributions, the *P*-values for predictions were calculated as: $p=\big(\#\big\{{\rho}_{\mathrm{null}}>{\rho}_{\mathrm{median}}\big\}+1\big)\!\big/ \!1001\big.$, where $\#\big\{{\rho}_{\mathrm{null}}>{\rho}_{\mathrm{median}}\big\}$ indicates the number of permutated predictions numerically greater than the median of the unpermutated predictions. As we expected a positive association between predicted and actual values, one-tailed *P*-values are reported. For the leave-one-group-out and external validation analyses, as they do not involve averaging prediction performance across different folds, we used Pearson correlation between actual and predicted cognitive phenotype to measure prediction performance. To determine if the diagnostic category has added information in the leave-one-group-out analysis, we randomly permuted group membership (200 times), keeping the relationship between behavioral phenotypes and CPM matrices intact. We maintained the same group size throughout permutations (i.e., the SCZ group remained *n* = 33, even though it was randomly filled with participants). The hypothesis was that if prediction performance in null distribution trials moved above/below the 97.5/2.5 percentiles, diagnostic category was detracting/contributing information from prediction success. False Discovery Rate (FDR) at *P* < 0.001 was used to correct for multiple comparisons.

### Mass Multivariate Analysis of Diagnostic Category

To measure differences in task connectomes among standard DSM-IV diagnostic categories, we used an extension of a traditional mass univariate, edge-wise analysis that uses multivariate approaches to combine all task connectomes in a single analysis, labeled Mass Multivariate Analysis (MMA) (Dadashkarimi et al. 2019). For each edge, connectivity strength from all task-based connectomes is included in a two-tailed MANOVA—the multivariate version of an ANOVA—resulting in a node-by-node matrix of *F*-values that represent the magnitude of differences across all diagnostic categories (i.e., healthy individuals and individuals with SCZ, BPAD, and ADHD) at each edge. We corrected for multiple comparisons using FDR at *P* < 0.05.

### Quantification of Node and Task Contribution

For the rCPM results, to quantify the contribution of each node to a given predictive model, we calculated the *n*-th node’s weight summed across all tasks and edges as: ${W}_n={\sum}_{k=1}^{268}{\sum}_{m=1}^6\boldsymbol{B}\big(k,m\big){\beta_m}^k\mathrm{std}\big({\boldsymbol{E}}_k\big(\kern-4pt:,m\big)\big)$, where $\boldsymbol{B}\big(k,m\big)$ indexes whether the $k\mathrm{th}$ edge is selected from the *m*$\mathrm{th}$ task, $std\big({\boldsymbol{E}}_k\big(\kern-3pt:,m\big)\big)$ represents the standard deviation of the *k*th edge in the *m*$\mathrm{th}$ task, and ${\beta_m}^k$ represents the weight learned by rCPM for the *k*th edge in the $m\mathrm{th}$ task. To quantify contribution at the network level, ${W}_n$ was averaged over each node in canonical functional networks, based on the functional networks presented in (Noble et al. 2017). To quantify the contribution of each task to a given predictive model, we calculated the $m$-th task’s average weight (labeled ${W}_m$) to the model as: ${W}_m={\sum}_{k=1}^{35\ 778}B\big(k,m\big){\beta_m}^k\mathrm{std}\big({\boldsymbol{E}}_k\big(\kern-6pt:,m\big)\big)$. To make the results more interpretable, ${W}_m$’s were then normalized to have sum 1, ${\sum}_{m=1}^6{W}_m=1$, so that it represents each task’s contribution proportion in the whole model. For the MMA results, node- and network-level contribution were quantified at the sum of all significant edges for a node or network.

### External Model Testing

We used a subset of the HCP (*N* = 514 subjects) to test the generalizability of our model. In this analysis, all subjects in one dataset (either CNP or HCP) were used for training and the other dataset is used for testing. The memory measures from the HCP and CNP are quite different with the HCP having fewer and less specialized memory tests than the CNP. As such, we were not able to theoretically combine the HCP measures in the same way as the CNP and chose to predict a general memory construct made of all memory measures available for each dataset. To do so, PCA was performed on all subjects’ memory scores in each dataset (HCP or CNP) independently to combine all memory measures into a “general memory” construct. Second, all task connectomes for an individual were combined using the general functional connectivity method (Elliott et al. 2019). The general functional connectivity method is a principled approach for creating a single connectome from disparate task fMRI data, via concatenating time courses from the different tasks. Like the memory measures, combining task-based connectomes prior to model building was chosen over the approach described above as the tasks from the HCP and CNP have no clear correspondence. Thus, assigning model weights for one set of tasks to another was not feasible. Third, using these general functional connectivity matrices, we trained a rCPM model of general memory using all subjects in the training dataset. Finally, we applied the trained model to the testing dataset to predict a general memory score. Pearson correlation between predicted and observed general memory was calculated to assess prediction performance. Both CNP and HCP were used as training and testing data.

### Network Overlap

To assess the overlap between edges that were predictive of each behavioral construct (i.e., in the 10-fold predictive model, where the salient statistic is a given node or network’s classifier weighting summed across all tasks and edges) or were significantly different across disease groups (i.e., in the mass-multivariate analyses, where the salient statistic is a given node or network’s *F*-score summed across all tasks and edges), we used three different approaches. First, correlation was used to compare the node-level contributions between predictive models and MMA results. Second, correlation was used to compare the network-level contributions between predictive models and MMA results. Third, we computed the probability that *n* shared edges exist between our networks and edges within or between 10 canonical functional networks. Significance was determined with the hypergeometric cumulative density function, which returns the probability of drawing up to *x* of *K* possible items in *n* drawings without replacement from an *M*-item population. This was implemented as follows: *p* = *1-hygecdf*(*x, M, K, n*) where *x* equals the number of overlapping edges, *n* equals the total edges in the first network of interest, *K* equals the total number of edges in the second network of interest, and *M* equals the total number of edges in the brain (35 778).

### Code and Model Availability

**Figure 2:**
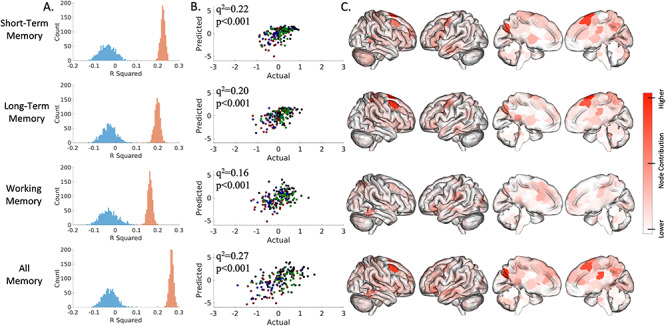
Connectome-based predictive model performance for transdiagnostic 10-fold cross-validation. The left column (*A*) shows a histogram of the model performance across 1000 iterations of the actual (red) and randomly permuted (blue) data. The middle column (*B*) shows how actual and predicted values compare for the median-performing model (green, SCZ; blue, BPAD; red, ADHD). The right columns (*C*) show surface plots of each node’s degree, which is defined as the number of edges per node that were weighted in 95% of iterations (the short-term memory model includes 289 consistently weighted edges; long-term, 276 edges; working, 174; all, 362). Leave-one-group-out analyses are presented in the Supplementary Materials.

Matlab scripts to run the main rCPM analyses can be found at https://github.com/YaleMRRC/CPM/tree/master/matlab/func. BioImage Suite tools used for analysis and visualization can be accessed at www.bisweb.yale.edu. Matlab scripts written to perform additional post hoc analyses are available from the authors upon request. The complete predictive model (based on the median-performing iteration, see Fig. 2) and a freely accessible instantiation of Bioimage Suite online wherein readers may access and navigate the entire model can be found at https://www.nitrc.org/projects/bioimagesuite/.

## Results

### Sample Characteristics

Demographic information of the CNP participants is shown in Supplementary Table 1. Additionally, the distributions of all memory measures as well as the latent memory constructs (i.e., principal components), broken down by clinical group, are shown in Supplementary Figures 2–4. There were no statistically significant differences in raw or latent memory constructs related to diagnostic category. Demographic information of the HCP participants can be found elsewhere (Greene et al. 2018).

### Transdiagnostic Prediction of Memory Constructs

We were able to predict working, short-, and long-term memory constructs across diagnosis. The six task-based connectomes predict working memory (median *q*^2^ = 0.16, *P* < 0.001, permutation testing, 1000 iterations, one-tailed), short-term (median *q*^2^ = 0.22, *P* < 0.001, permutation testing 1000 iterations, one-tailed), and long-term (median *q*^2^ = 0.20, *P* = < 0.001, permutation testing, 1000 iterations, one-tailed). Similar prediction accuracy is observed if all memory measures—regardless of category—are included (median *q*^2^ = 0.27, *P* < 0.001, permutation testing, 1000 iterations). We conduct multiple post hoc follow-up analyses to assess the robustness of our results. We test the effect of sample size, motion, number of edges, and edge selection threshold on model performance in Supplementary Figures 5–9.

In line with the previous CPM results, our models are complex with contributions from each task and distributed across multiple brain areas. In general, each task-based connectome contributes to prediction performance (Supplementary Fig. 10). For short-term and long-term memory, the PAM-RET and BART tasks contributed the most to overall prediction. For working memory, task contributions are more uniform. For the short- and long-term memory models, the top three contributing nodes to prediction were located in the right prefrontal cortex, cerebellum (left crus I), and the right motor strip (Fig. 2*C*). For the working memory model, the top three contributing nodes are in the left medial prefrontal, right temporal–parietal junction, and right temporal lobe (Fig. 2*C*). Finally, all models were able to predict the other memory constructs (Supplementary Fig. 11). In other words, models trained with either working, short-, or long-term memory also predicted the other memory measures not used for training.

### Leave-One-Group-Out Predictive Models of Memory

We are able to predict memory performance across diagnostic groups (see Supplementary Fig. 12). In 14 of 16 analyses, models—trained in all but one group—predict working, short-term, and long-term memory in the left-out group. This is true even when models are trained only on patients and tested on healthy controls. Only the short- and long-term memory models are created with BPAD as the left-out group was not significant. When permuting diagnostic category labels to test whether diagnosis adds value to the model, we did not observe a clear change in model performance across the true and 200 randomly permuted diagnosis permutations, indicating that diagnostic category does not contribute information to prediction performance.

### Mass Multivariate Analysis of Diagnostic Category Differences

**Figure 3:**
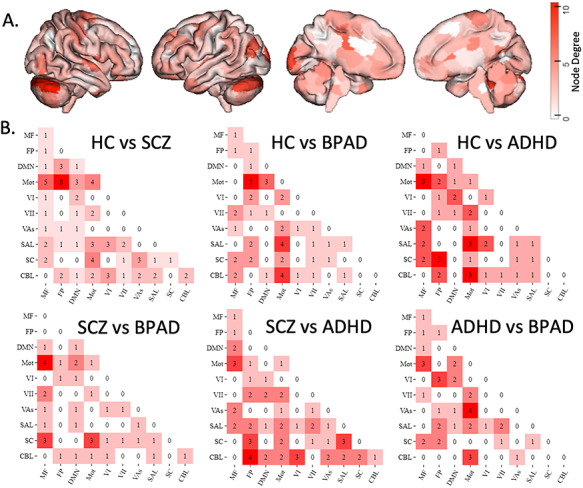
Mass multivariate analysis of disease group differences in brain network structure across all tasks. (*A*) Surface illustration of nodes where edges (network connections) significantly differ across all clinical groups, as measured with Hotelling’s T2. (*B*) illustrates significant network-to-network edges. (Circle plots showing nonsummarized edges can be referenced in the Supplementary Materials; Network Labels: MF, medial frontal; FP, frontoparietal; DMN, default mode; Mot, motor cortex; VI, visual A; VII, visual B; VAs, visual association; SAL, salience; SC, subcortical; CBL, cerebellum).

We find differences between each diagnostic category’s task-based functional connectomes, indicating that, while psychiatric diagnoses do not contribute to memory deficit prediction, there are group differences in connectivity. After strict network-based multiple comparison correction, only 368 edges show group differences, representing only 0.17% of all possible edges. The cerebellum appears to be overrepresented in connection strength differences across the disease group as it contained the top three nodes (right V, right crus I, left crus I) in terms of the number of edges (Fig. 3). Pairwise comparisons between groups suggest that group differences are largely found in medial frontal, frontoparietal, cerebellar, and motor networks. Unthresholded edge locations are illustrated in Supplementary Figure 13.

### Network Profile and Overlap

**Figure 4:**
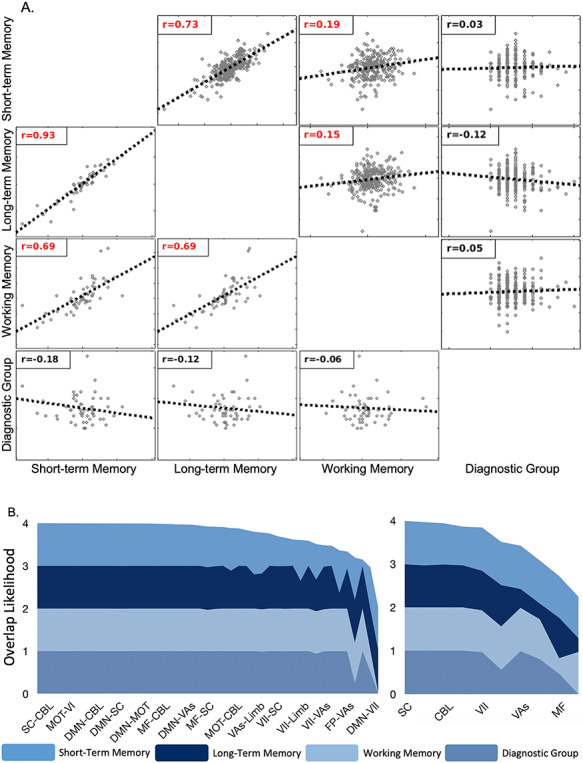
Similarity between the short, long, and working memory 10-fold predictive models and the diagnostic group mass multivariate analyses. (*A*) Correlation matrices for the node- or network-level contribution to the three predictive models and the node- or network-level *F*-score of the mass multivariate analyses. The upper triangle shows node-level correlations; the lower triangle shows network-level correlations. Red font indicates significant correlations. Overall, all memory models were correlated with each other but not the MMA results. (*B*) Layer thickness represents the likelihood that a particular internetwork (Left) or intranetwork (Right) edge is selected by the model, as computed by the hypergeometric distribution. Ridge regression analyses are indicated by short, long, and working memory. MMAis indicated by disease group. Each layered plot shows the cumulative (sum) likelihood (1.0—*P* value) estimated from the probability of edges being shared between a priori networks and the short-, long-, and working memory models (Fig. 2) and groups differences associated with diagnostic categories (Fig. 3). Networks and internetwork pairs are ordered from greatest to least cumulative likelihood. Only the most overlapping networks are shown for simplicity.

We find that models for short-term, long-term, and working memory were significantly correlated with each other at the node level and network level (Fig. 4*A*). The short- and long-term memory models were more similar to each other than they were to working memory models. In contrast, the models for short-term, long-term, and working memory were not correlated with MMA networks. Finally, we find that similar networks are overrepresented in the three memory models and MMA of diagnostic categories (Fig. 4*B*). Edges in the cerebellum and subcortical networks and edges between the cerebellum, subcortical, default mode, and motor networks are more likely to be in these results than edges in other networks. Together, these results suggest that, while they involve similar large-scale networks, the memory models and MMA results are distinct.

### Model Validation on External Datasets

The general memory model (including working, short- and long-term memory measures) trained on the CNP dataset (*N* = 172) successfully generalized to the HCP dataset (*r* = 0.17, *P* < 0.01, df = 513). At the same time, a summary memory model trained on the HCP dataset (*N* = 514) generalized back to the CNP dataset (*r* = 0.40, *P* < 0.01, df = 170). We observe differences in prediction performance when training with the CNP and HCP datasets. We suspect that this is because the sample size used to train our models is three times large for the HCP compared to the CNP (i.e., 517 vs. 175), allowing us to achieve higher prediction performance when training with the HCP.

## Discussion

Using large, publicly available datasets and advanced predictive modeling, we show that the macroscale circuitry of different memory constructs (working, short-, and long-term memory) are transdiagnostic. In other words, individuals with SCZ, BPAD and ADHD show a common connectivity pattern which is predictive of memory performance. This pattern is distributed throughout the brain and includes regions in the subcortex, default mode network, limbic network, and cerebellum. These markers of memory generalize across diagnostic categories despite statistically significant differences in whole-brain functional connectivity patterns observed with our MMA. Finally, we show that connectome-based models of memory generalize across independently collected datasets. Overall, our results suggest that differences in memory across individuals with and without psychiatric disorders arise from the same underlying connectivity networks.

Although patients with mental illness have historically been viewed as neurobiologically discrete categories, our results are consistent with a growing body of work which supports a diagnostic continuum of cognition that includes healthy controls (Cuthbert and Insel 2013; Yamashita et al. 2018). For the three memory constructs we evaluate (working, short-, and long-term memory), we show that models built from patients diagnosed with SCZ, BPAD, and ADHD predict memory function in healthy control subjects. This indicates that a similar macroscale brain network subserves memory in patients with various diagnostic categories (Insel et al. 2010; Insel and Sahakian 2012).

Memory is but one of many facets of cognition affected by mental illness (Millan et al. 2012). There is reason to suspect that other aspects of cognition can be predicted by shared macroscale brain networks across diagnostic group. For example, models of attention generalize across healthy controls and individuals with ADHD (Rosenberg et al. 2016), and models of social impairment generalize across healthy controls, individuals with ADHD, and individuals with autism (Lake et al. 2019). It further seems possible that high-level summary measures of cognition such as “gF” (Greene et al. 2018), or of psychopathology such as “P,” (Caspi and Moffitt 2018) could be represented by shared macroscale brain networks across diagnostic group. Overall, future work could test whether other cognitive measures can be predicted in a transdiagnostic fashion.

Nevertheless, it remains important to test for specificity of a model for an aspect of cognition, in addition to generalization. We showed that, while the short- and long-term memory models appear to have distinct node-level features from the working memory model, all models predicted the other memory constructs well, suggesting that connectome-based predictive models may not be sensitive enough to resolve the distinction between these constructs. As other experimental evidence suggests that memory shares overlapping circuitry with attention, decision-making, self-regulation, problem solving, and language (Etkin et al. 2013), future work would be to test the specificity of our memory model against these aspects of cognition. Preliminary evidence suggests that connectome-based predictive models can be specific to a phenotype. For example, models of different components of attention appear to be specific to that component and do not predict other components (Rosenberg, Hsu, et al. 2018b).

We further show differences in the brain’s overall functional organization as a function of diagnostic category. While the memory prediction models and the MMA results were distinct (see Fig. 4*A*), there were similarities in which networks were overrepresented (e.g., the cerebellum; see Fig. 4*B*). Therefore, although diagnosis-related pathophysiologies are associated with significant changes in the brain’s overall functional organization, possibly involving similar large-scale networks, these changes do not fundamentally alter the functional anatomy of memory. That we do not observe compensatory circuits engaged to maintain cognitive performance in mental health may be important. This suggests that the plasticity of the brain constrains recovery or, perhaps, is part of the problem (i.e., reduced neuroplasticity). However, future work is needed to test this idea.

Given the phenotypic variability and cognitive complexity of memory, it is unsurprising that our functional connectome-based models reflect diffuse cortical networks. Functional connectome-based models of cognitive traits tend to be very complex, and as many as 214 668 edges may be used as an initial input to a given model. While we use a comparatively small subset of the possible edges to ultimately define the model, the contributing edges typically number in the hundreds. Although each individual edge or task-based connectome is either weakly predictive or not predictive at all, combining edges *en masse* across multiple task-based connectomes improves the prediction of behavioral traits (Gao et al. 2019). We extend the observation that different tasks contribute differentially to the final predictive model, further suggesting that the battery of tasks used in prediction is an important consideration in clinically relevant models (see Supplementary Fig. 5).

This could be an important insight into biomarker development: successful models of brain–behavior relationships are not necessarily simple and, given that the human brain and behavior are highly complicated, models could very well be necessarily complex (Burnham and Anderson 2004; Rosenberg, Casey, et al. 2018). Although communicating a complex model (i.e., in a paper or figure or conversation) requires a reduction of complexity (Yip, et al. 2019), this can appear to come at the cost of learning something neurobiologically meaningful (Bzdok et al. 2019).

With this in mind, our results implicate the importance of the cerebellum. Consistently, the bilateral crus I was a key node across analyses. This cerebellar region is part of a nonmotor gradient in the cerebellum that is engaged in working memory tasks, (Guell et al. 2018) consistent with recent parcellations that split the cerebellum into sensorimotor, cognitive, and limbic functions (Schmahmann and Caplan 2006; Riedel et al. 2015). Furthermore, recent evidence implicates the cerebellum as part of a whole-brain network affected in psychiatric disease (Ji et al. 2019), consistent with our results. Together, these results add to the improving understanding of the cognitive components of the cerebellum (Buckner 2013).

This study has several strengths, including use of an advanced, whole-brain predictive modeling approach that takes advantage of complementary information from several sources, leave-one-group-out transdiagnostic modeling, and out-of-sample replication. However, several limitations should be noted. While the CNP sample is large (*N* = 172), the number of individuals in each diagnostic category is relatively modest (smallest group, *N* = 30); thus, further work in larger samples is warranted. In addition, the functional significance of the identified networks in relation to other aspects of cognition and mental health remains to be determined. While we have attempted to control for potential confounds, we cannot entirely exclude the effects of other clinical variables, such as medication, disease severity, and general cognitive impairment, on connectivity strength. To facilitate replication, we have shared all analysis software and models (see Code and Model Availability).

In conclusion, we have used CPM to define the macroscale brain networks that predicts memory function across diagnostic categories. We have further showed that, notwithstanding differences in functional connectivity between diagnostic groups, models trained on patients with mental illness generalize to healthy controls. We further show that predictive models of memory function generalize across independent datasets. Together, these observations suggest that the same macroscale brain networks subserve memory across diagnostic groups and that individual differences in memory performance are related to individual differences within this brain circuit.

We recommend that brain models of memory should be extended in a larger, more diverse transdiagnostic sample.

## Supplementary Material

Manuscript_SUPMAT_20201111_bhaa371

## Notes

The authors thank Kristin S. Budde (MD MPH, Yale University and University of Washington) for her editorial assistance, Karen E. Blackmon (PhD, St. George’s University and Mayo Clinic) for her help understanding neuropsychologic tests, and Jeetu Bhawnani (MS, Yale University) for his IT support. *Conflict of Interest*: None of the authors have conflicts of interest relevant to this manuscript.
