# Supplementary material for: Transdiagnostic, Connectome-Based Prediction of Memory Constructs Across Psychiatric Disorders
Source: Cereb Cortex. 2020 Dec 21;31(5):2523–33. doi: 10.1093/cercor/bhaa371 (PMC8023861; doi:10.1093/cercor/bhaa371)
Supplement: Manuscript_SUPMAT_20201111_bhaa371 [file manuscript_supmat_20201111_bhaa371.docx]

**SUPPLEMENTARY METHODS**

**CNP Imaging parameters:** Brain imaging data were acquired on one of two 3T Siemens Trio scanners at UCLA. Functional MRI data were collected using a T_2_*-weighted echoplanar imaging (EPI) sequence (TR=200ms, TE=30ms, flip angle=90°, slice thickness=4mm, oblique slice orientation).

**HCP Imaging parameters:** HCP S900 Release Data were obtained from the HCP 900-participant release of December 2015. From the larger dataset, we restricted our analyses to those individuals who participated in all nine fMRI conditions (seven task, two rest), whose mean frame-to-frame displacement was less than 0.1 mm and whose maximum frame-to-frame displacement was less than 0.15 mm, and for whom memory measures were available (n = 515; 241 males; ages 22–37). All fMRI data were acquired on a 3 T Siemens Skyra using a slice-accelerated, multiband, gradient-echo, EPI sequence (TR = 720 ms, TE = 33.1 ms, ﬂip angle = 52°, resolution = 2.0 mm^3^, multiband factor = 8). Images acquired for each subject include a structural scan and eighteen fMRI scans (working memory (WM) task, incentive processing (gambling) task, motor task, language processing task, social cognition task, relational processing task, emotion processing task, and two resting-state scans; two runs per condition (one LR phase encoding run and one RL phase encoding run)) split between two sessions.

**Functional parcellation and network definition.** We used the Shen 268-node atlas to parcellate the fMRI data into functionally coherent nodes (Shen, Tokoglu, Papademetris, & Constable, 2013). The Shen 268-node atlas is derived from an independent data set using a group-wise spectral clustering algorithm. The mean time courses of each node pair were correlated, and correlation coefficients were Fisher transformed, generating six 268 × 268 connectivity matrices per subject for each fMRI run; therefore, each individual had a total of 214,668 unique edges. The same spectral clustering algorithm was used to assign these 268 nodes to 8 networks (Finn et al., 2015; Shen et al., 2017), and the subcortical-cerebellar network was split into networks 8–10 (Noble et al., 2017). These networks are named based on their approximate correspondence to previously defined resting-state networks, and are labeled according to the following scheme: MF = medial frontal, FP = frontoparietal, DMN = default mode, Mot = motor cortex, VI = visual A, VII = visual B, VAs = visual association, SAL = salience, SC = subcortical, CBL = cerebellum.

**Effects of motion.** Motion is an important confound for estimates of functional connectivity. In our analysis, motion was significantly correlated with most individual behavioral measures (see Supplementary Figure 9). We used two methods for accounting for this confound: we excluded all subjects with >0.15 mm grand mean frame-to-frame motion across all tasks and we regressed out motion during the feature-selection step (see Figure 1) using partial correlation (i.e. as opposed to correlation). (**Hsu W** et al. 2018) All results presented in the main text were performed using the partial correlation step, however this did not substantially change model performance (see Supplementary Figure 6).


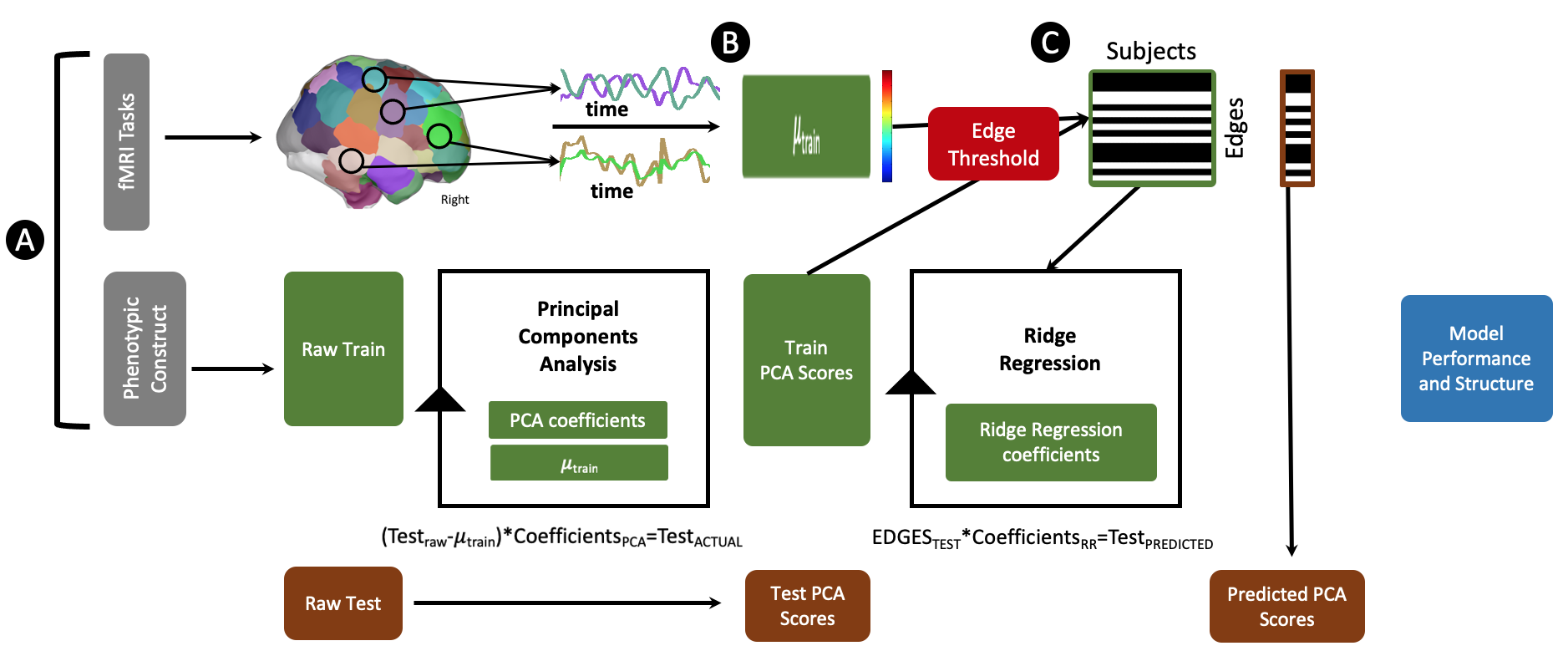


**Supplementary Figure 1. Overview of Supplementary Analyses**. (A) In Supplementary Figure 3, we show the effect of sample size on model performance for the 10-fold phenotype predictive analyses. (B) We evaluated the effect of pre-threshold edge number on model performance, shown in Supplementary Figure 6. (C.1) We evaluated the effect of different statistical significance thresholds on the correlation analysis, we ran the analyses at p<0.01, p<0.005, and p<0.001 thresholds for the k-10, shown in Supplementary Figure 7. (C.2) We evaluated the effect of head motion on prediction performance by running analyses with and without motion regression (e.g. partial correlation, as reported in Figure 2), shown in Supplementary Figure 8. Results did not substantially change.

|  | **Included Number** | **Men** | **Women** | **Age +/- Stdev** | **Years Education** | **Prescribed Antipsychotics** | **Prescribed Antidepressants** | **Prescribed Mood Stabilizers** |
| --- | --- | --- | --- | --- | --- | --- | --- | --- |
| **Controls** | **73** | **38** | **35** | **30 +/-8** | **15 +/-1** | **0** | **0** | **0** |
| **SCZ** | **33** | **24** | **9** | **34 +/-9** | **13 +/-1** | **27** | **10** | **7** |
| **BPAD** | **34** | **17** | **17** | **35 +/-9** | **15 +/-2** | **18** | **11** | **23** |
| **ADHD** | **32** | **17** | **15** | **31 +/-10** | **15 +/-2** | **1** | **3** | **1** |
| **TOTAL** | **172** | **96** | **76** |  |  | **46** | **24** | **31** |

**SUPPLEMENTARY RESULTS**

**Table S1.** Demographic Information for the CNP dataset.


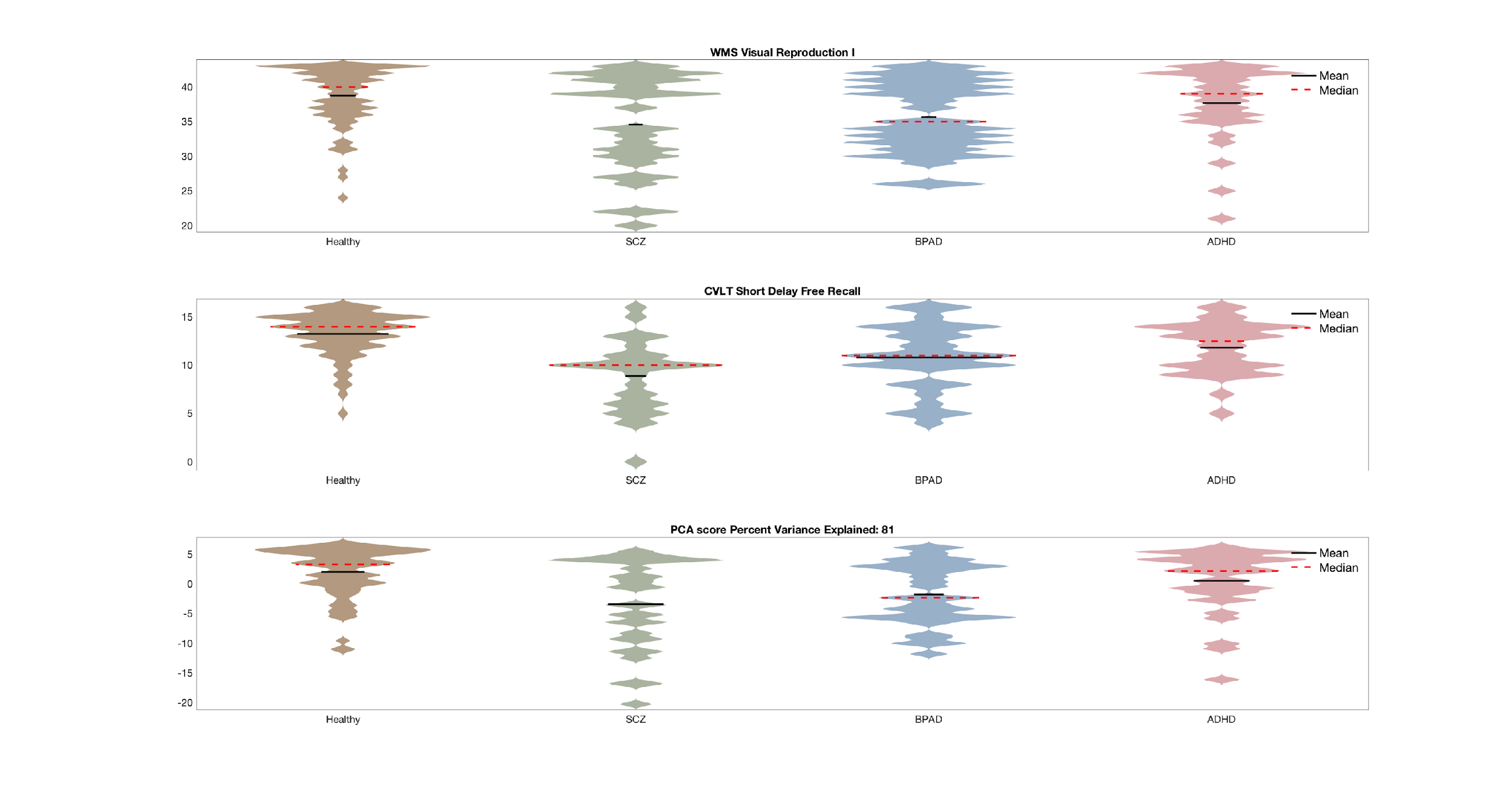


**Supplement Figure 2: Short-Term Memory Phenotype Characteristics.** The top row shows boxplots of the individual behavioral measures (as titled) organized by clinical group. These behavioral measures were normalized and combined through principal components analysis. The bottom row shows the percent of variance explained by each of the principal component scores (left) and a violin plot of the first principal component score for each clinical group (right). CVLT=California Verbal Learning Task.


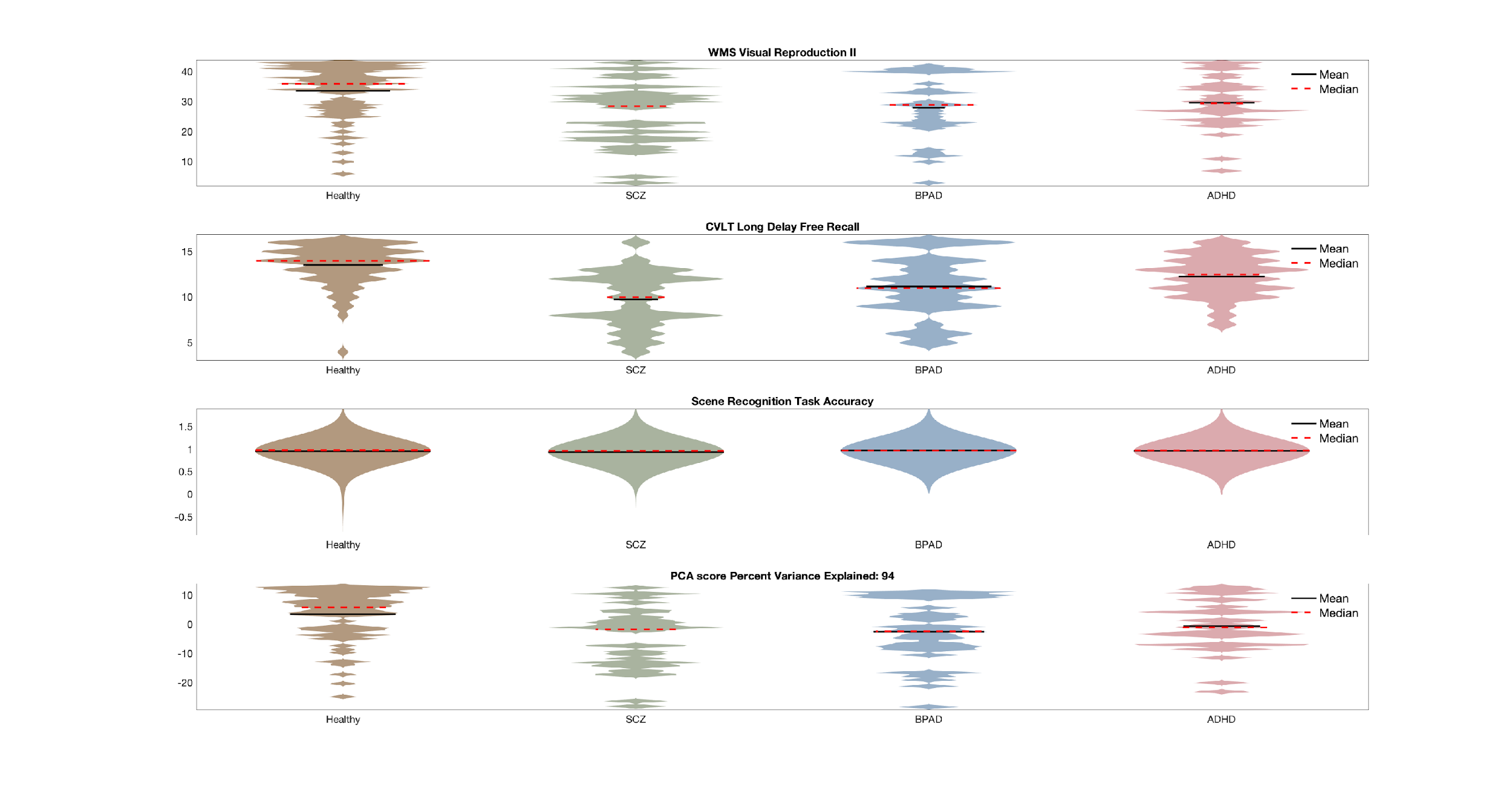


**Supplementary Figure 3. Long-Term Memory Phenotype Characteristics.** The top row shows violin plots of the individual behavioral measures (as titled) organized by clinical group. These behavioral measures were normalized and combined through principal components analysis. The bottom row shows the percent of variance explained by each of the principal component scores (left) and a boxplot of the first principal component score for each clinical group (right). CVLT=California Verbal Learning Task.


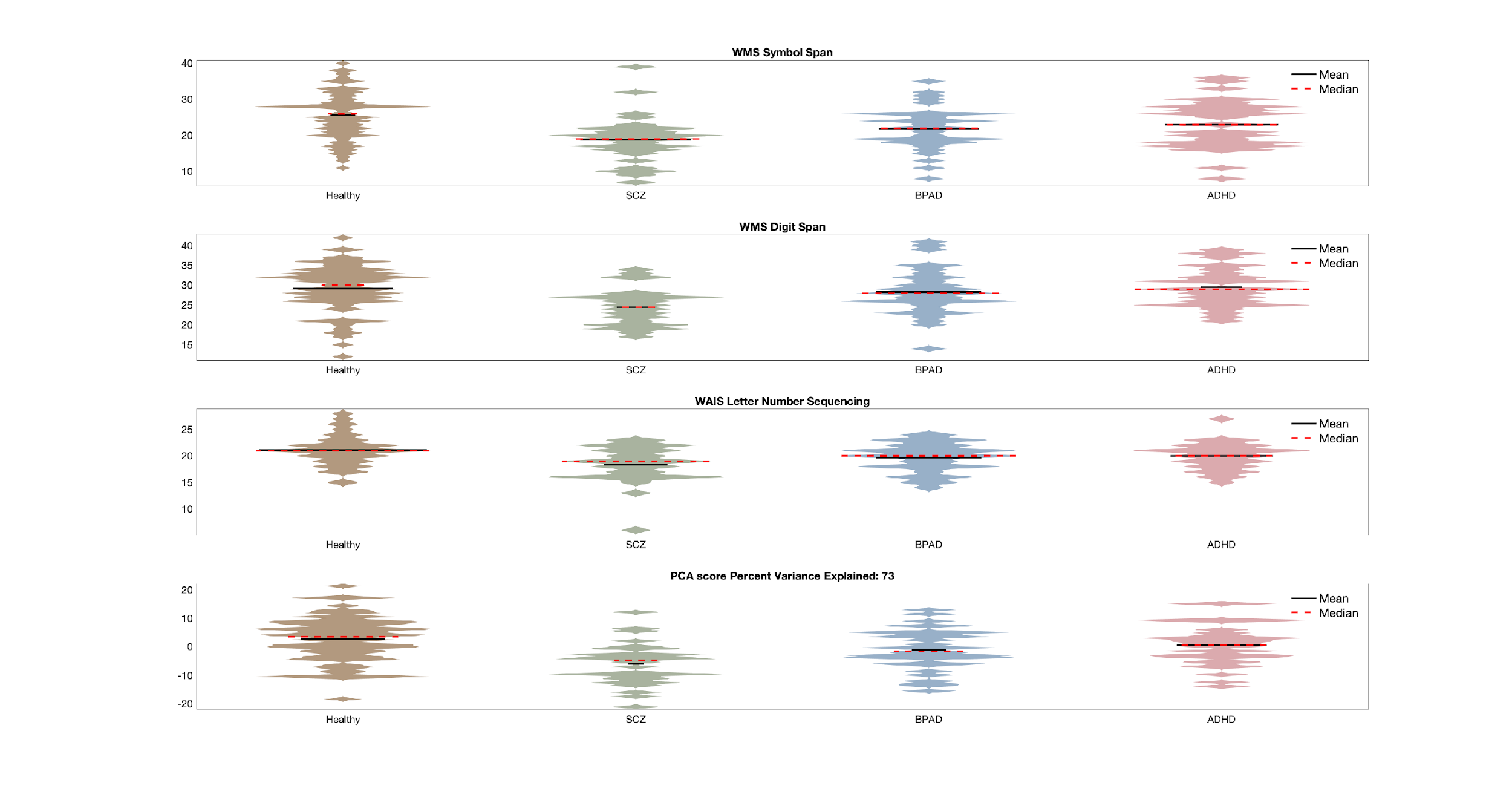


**Supplementary Figure 4. Working Memory Phenotype Characteristics.** The top row shows boxplots of the individual behavioral measures (as titled) organized by clinical group. These behavioral measures were normalized and combined through principal components analysis. The bottom row shows the percent of variance explained by each of the principal component scores (left) and a boxplot of the first principal component score for each clinical group (right). WMS=Weschler Memory Scale; WAIS=Weschler Adult Intelligence Scale.


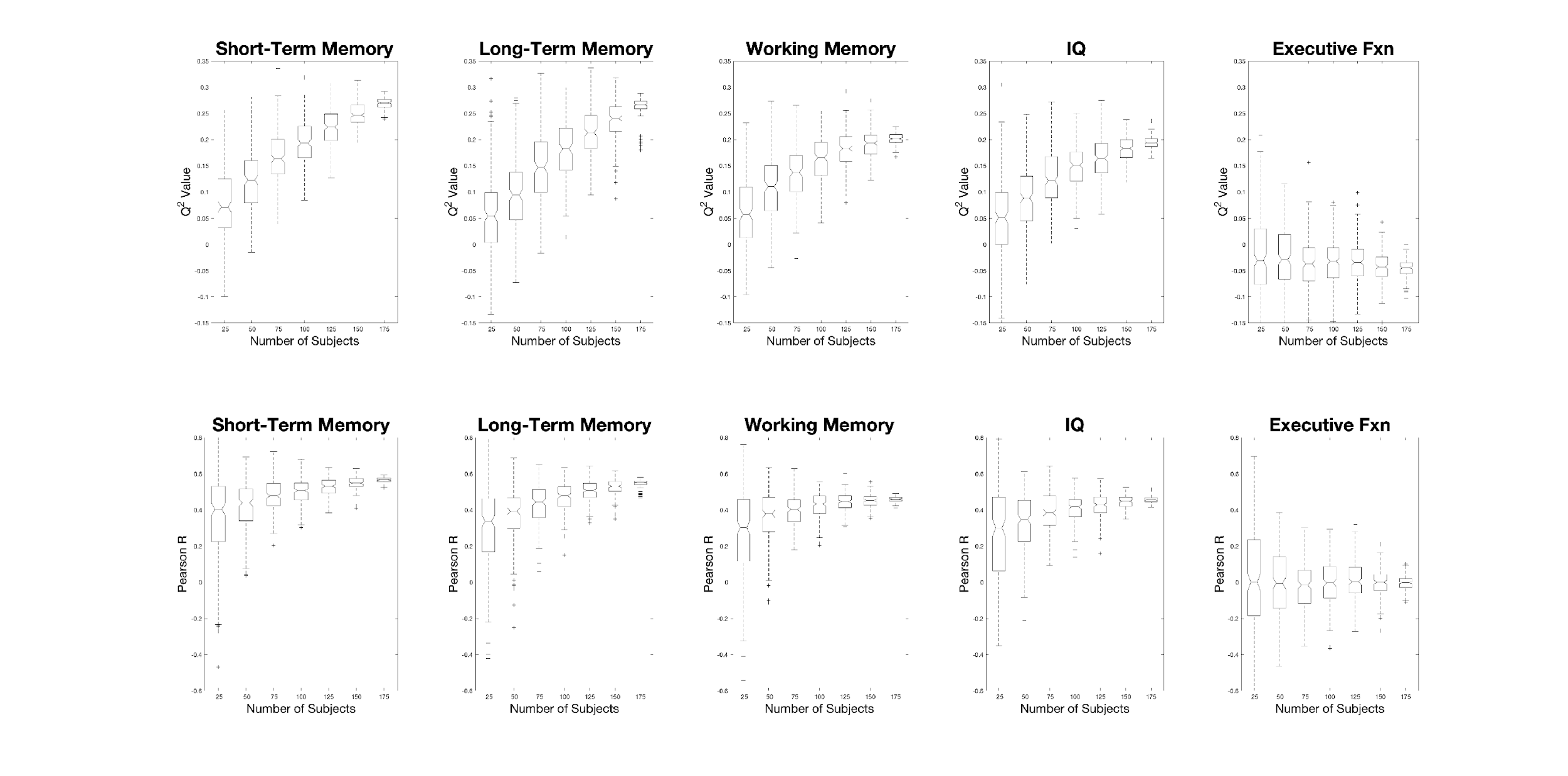


**Supplementary Figure 5.** Performance as a function of sample size for 10-fold ridge regression analysis. We iterated these analyses 200 times to produce the error bars. We did not regress out motion in these analyses.


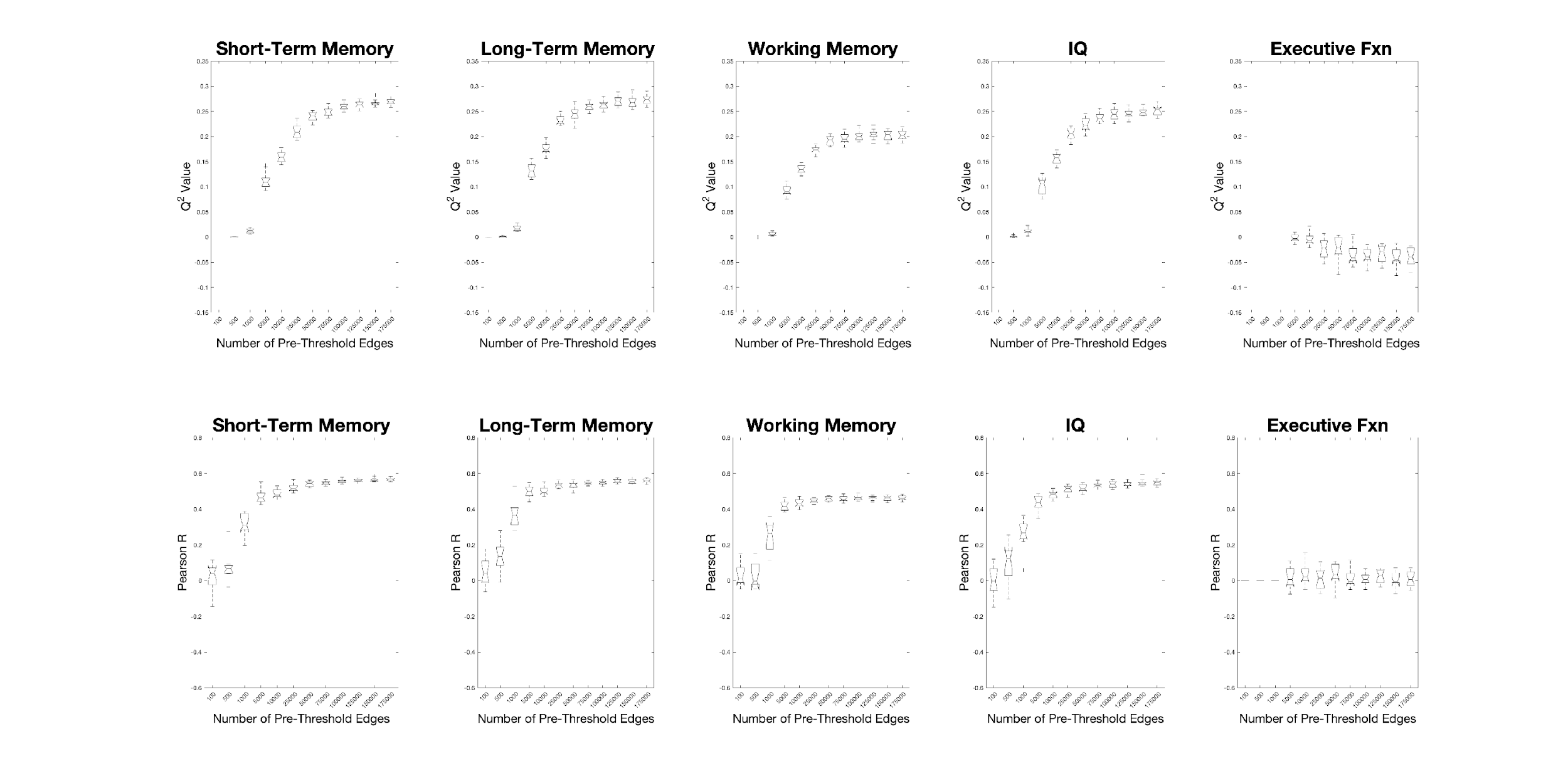


**Supplementary Figure 6.** Performance as a function of pre-thresholded edge number for 10-fold ridge regression analysis. All edge increments were randomly selected from the total number. Top row shows performance measured with Q-Squared. Bottom Row shows performance measured as Pearson correlation between actual and predicted phenotype scores. We iterated analyses 200 times to produce error bars; we did not regress out motion in these analyses.


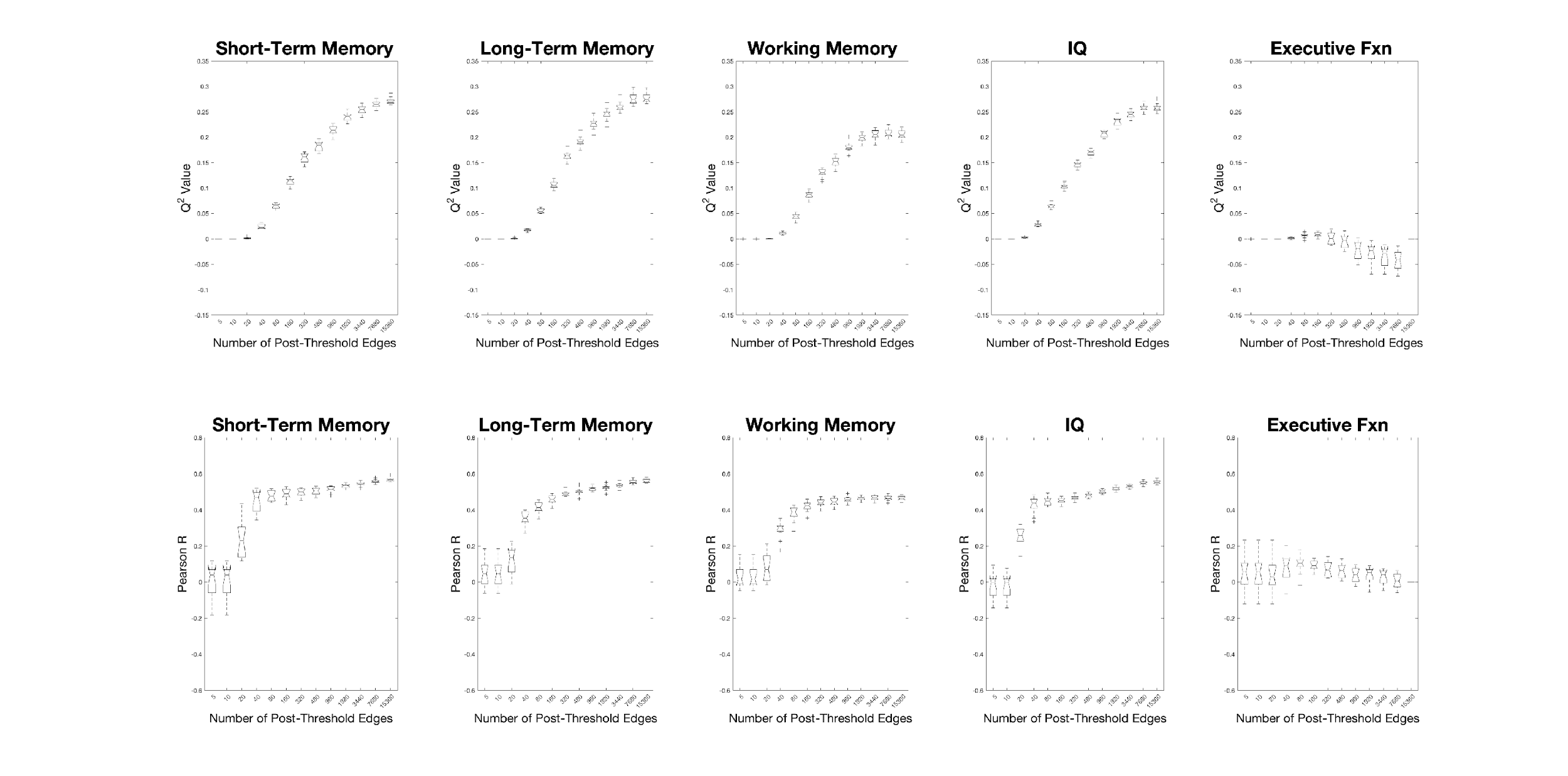


**Supplementary Figure 7.** Performance as a function of edge threshold for 10-fold ridge regression analysis. Each increment selected the most significant (smallest p-value) 5, 10, etc. edges. Top row shows performance measured with Q-Squared. Bottom Row shows performance measured as Pearson correlation between actual and predicted cognitive construct scores. We iterated analyses 200 times to produce error bars; we did not regress out motion in these analyses.


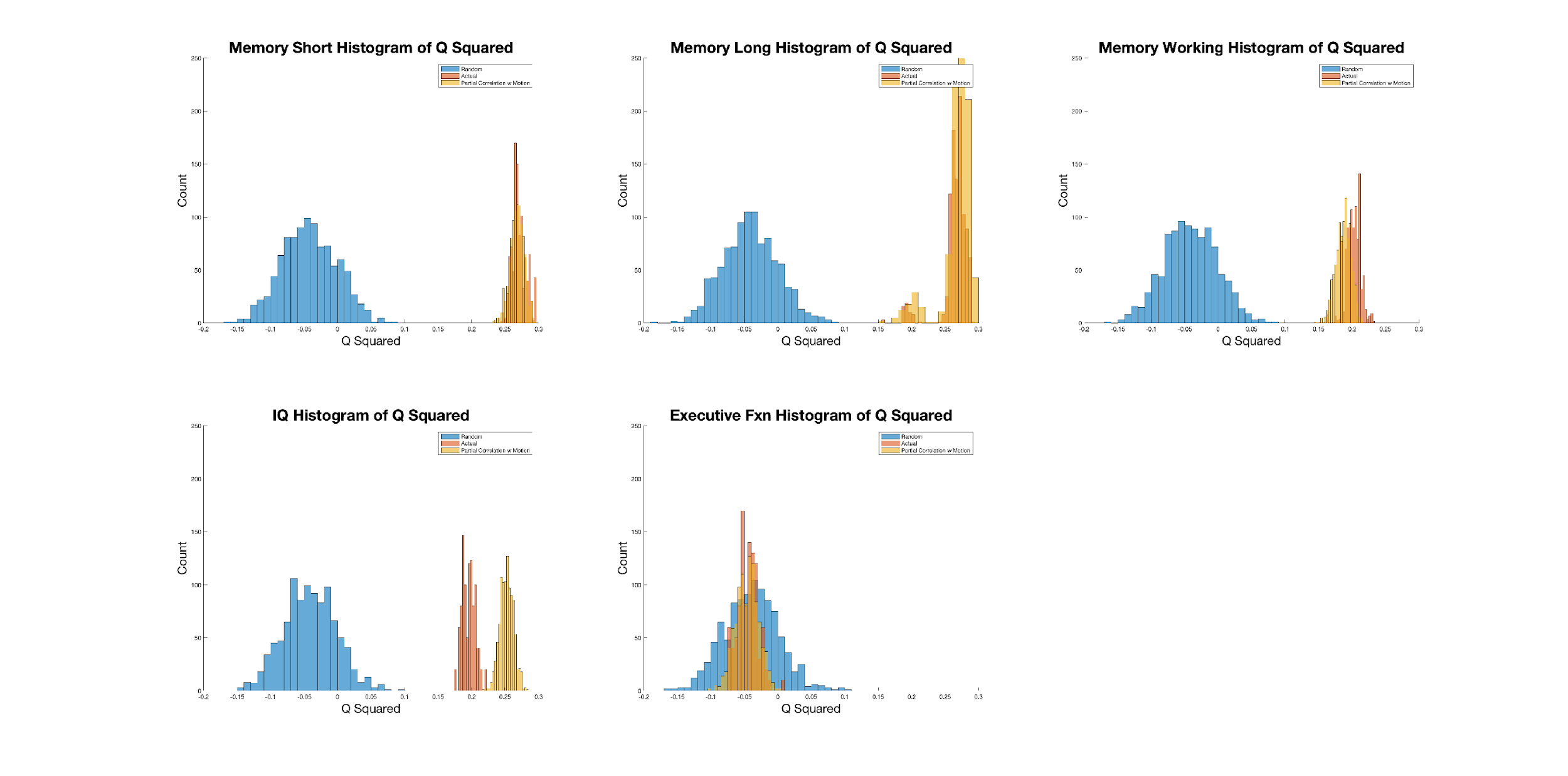


**Supplementary Figure 8.** The effect of motion on model performance. We ran the k-10 fold analyses 1,000 times without partial correlation for motion. There was no notable difference in performance compared to the partial correlation analysis with motion.


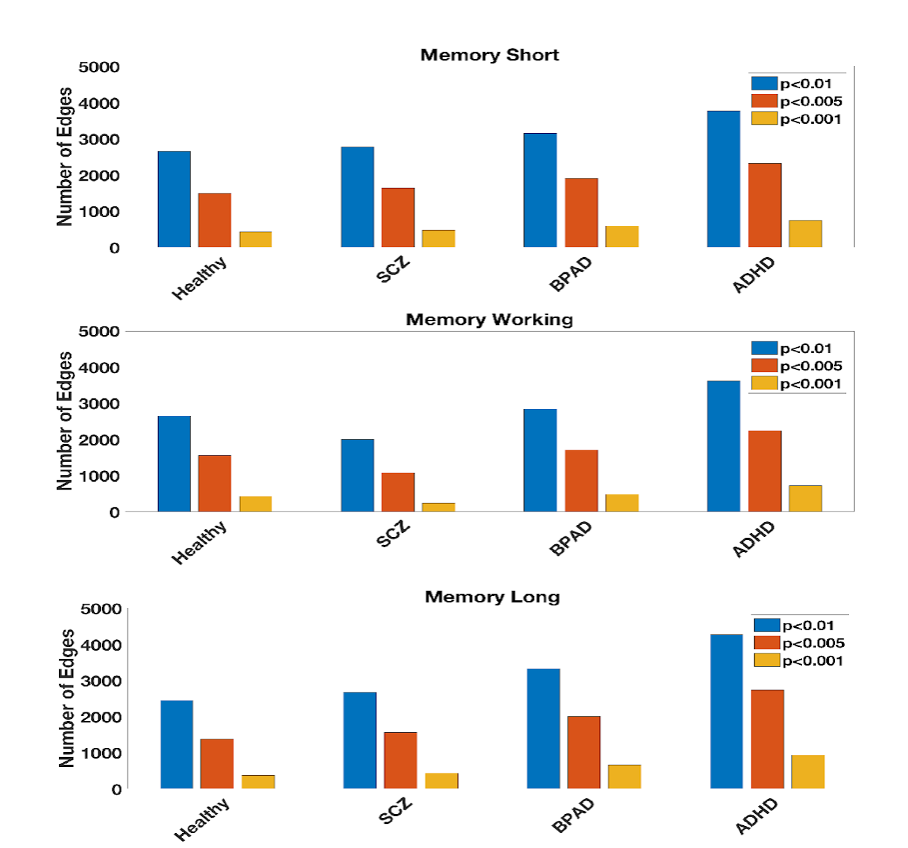


**Supplementary Figure 9. Number of edges as a function of edge threshold.** The effect of different thresholds on the number of significant edges used in ridge regression analyses. As expected, as the threshold becomes more conservative, fewer edges are used in final predictive analysis.


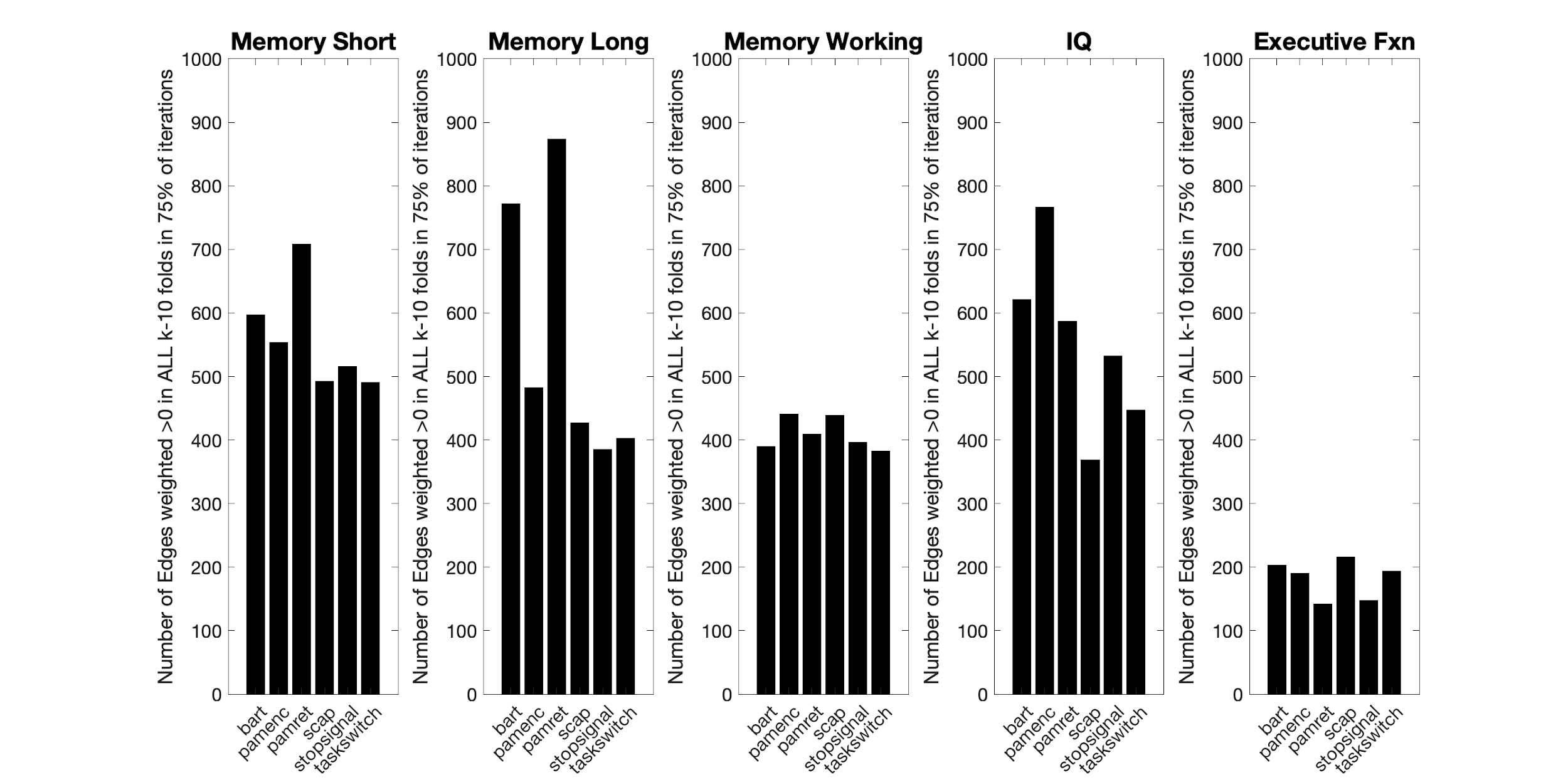


**Supplementary Figure 10. Breakdown of weighted edges by task.** For each task, this figure shows how many edges were weighted by the ridge regression algorithm in all 10 folds of 75% of the 1,000 iterations.


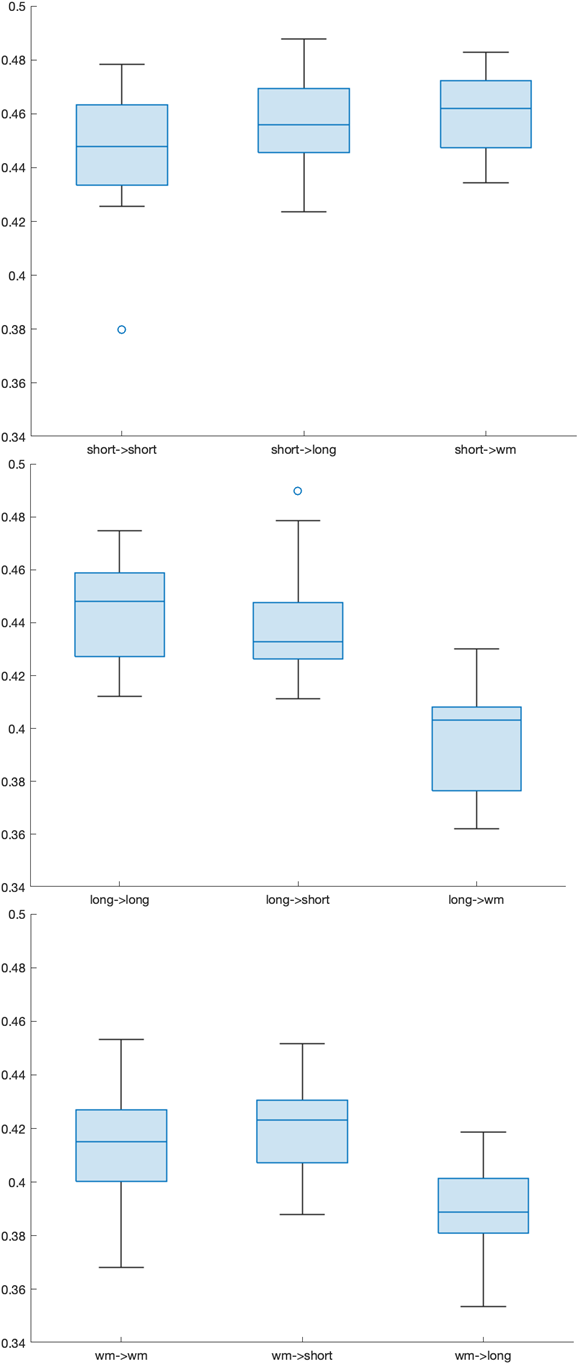


**Supplementary Figure 11. Cross model prediction.** Each of the three models of memory successfully predicted the other memory measures. In other words, models trained with either working, short-, or long-term memory also predicted the other memory measures not used for training. There were no differences between predicting the different memory types.


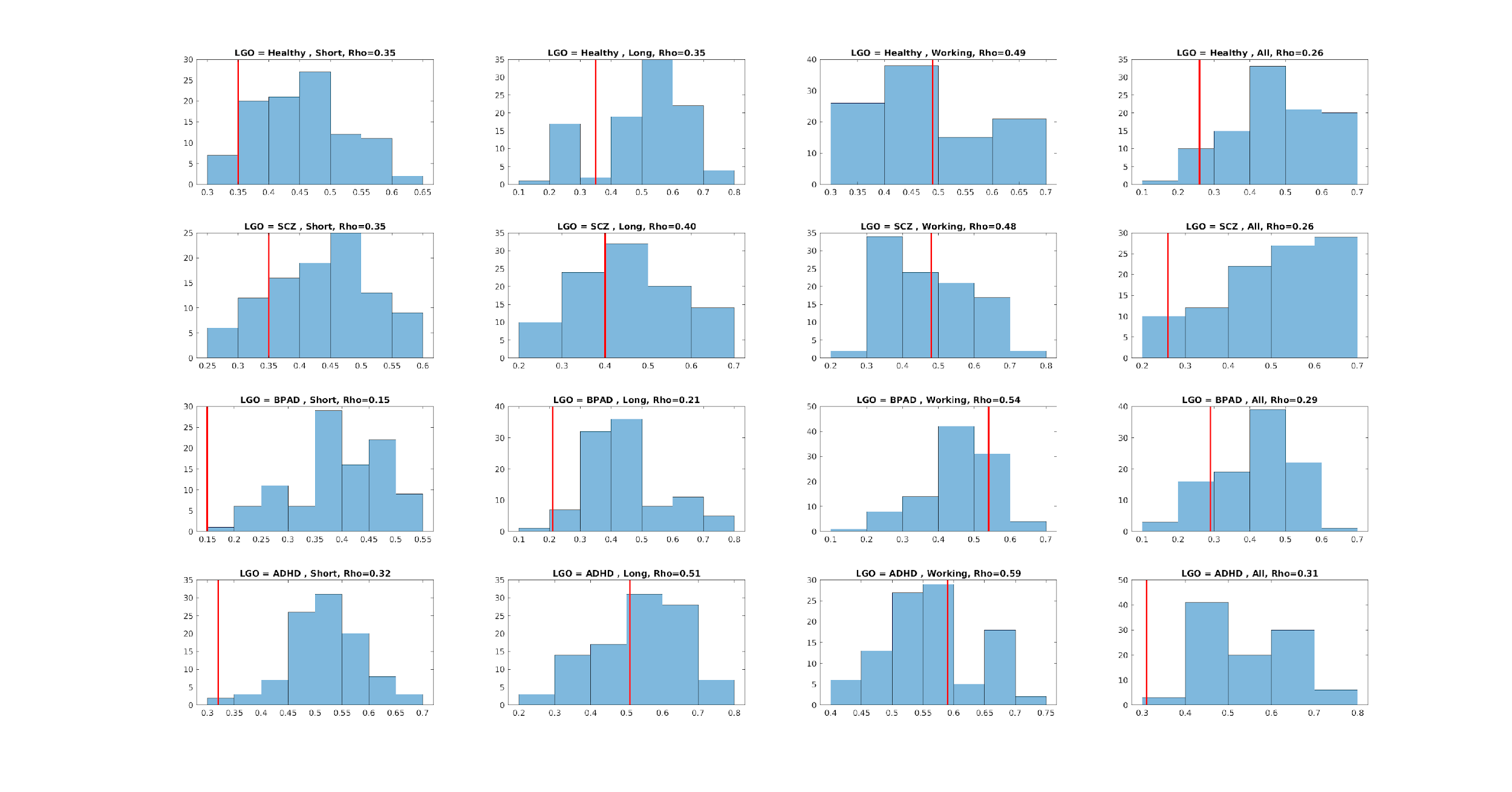


**Supplementary Figure 12. Leave-group-out model performance.** Models were trained on all data except for the test group (organized by row). Model performance in test group indicated by red line. Group labels were randomly combined to create a null distribution, shown in blue.


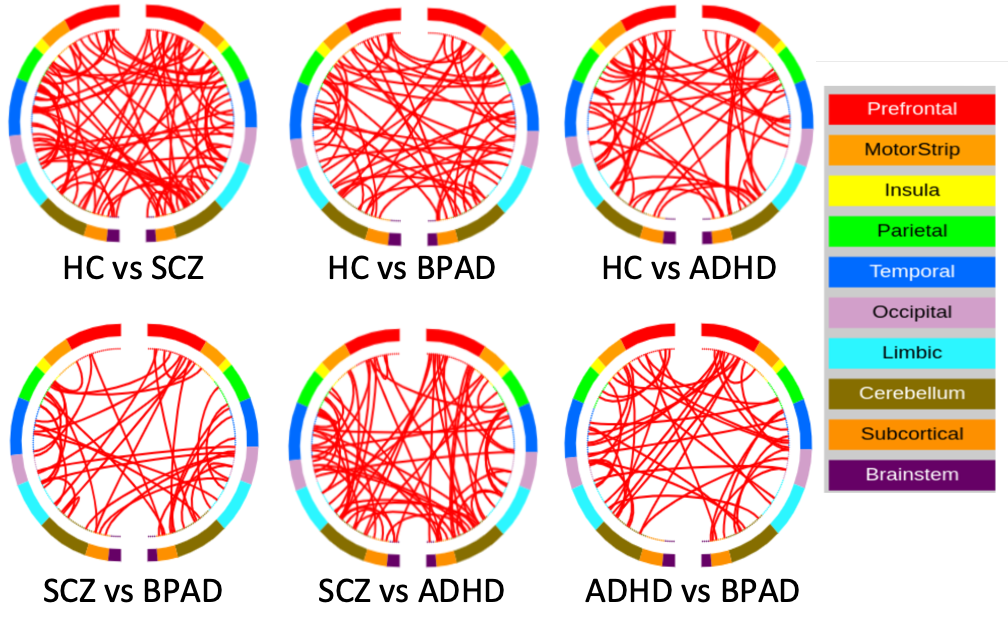


**Figure 13. Mass multivariate analysis of disease group difference in brain network structure across all tasks.** Circle plots illustrate the spatial locations of all edges exhibiting significant group differences that were not thresholded by degree or summarized by networks.

Finn, E. S., Shen, X., Scheinost, D., Rosenberg, M. D., Huang, J., Chun, M. M., et al. (2015). Functional connectome fingerprinting: identifying individuals using patterns of brain connectivity. *Nature Publishing Group*, *18*(11), 1664–1671. http://doi.org/10.1038/nn.4135

First, M. B., Spitzer, R. L., Gibbon, M., & Williams, J. (2002). Structured clinical interview for DSM-IV-TR axis I disorders, research version, patient edition.

Glasser MF, Sotiropoulos SN, Wilson JA, Coalson TS, Fischl B, Andersson JL, Xu J, Jbabdi S, Webster M, Polimeni JR, Van Essen DC, Jenkinson M, Consortium W-MH. The minimal preprocessing pipelines for the Human Connectome Project. Neuroimage. 2013;80:105-124.

Gorgolewski, K. J., Auer, T., Calhoun, V. D., Craddock, R. C., Das, S., Duff, E. P., et al. (2016). The brain imaging data structure, a format for organizing and describing outputs of neuroimaging experiments. *Scientific Data*, *3*, 160044. http://doi.org/10.1038/sdata.2016.44

Hsu, W.-T., Rosenberg, M. D., Scheinost, D., Constable, R. T., & Chun, M. M. (2018). Resting-state functional connectivity predicts neuroticism and extraversion in novel individuals. *Social Cognitive and Affective Neuroscience*, *13*(2), 224–232. http://doi.org/10.1093/scan/nsy002

Noble, S., Spann, M. N., Tokoglu, F., Shen, X., Constable, R. T., & Scheinost, D. (2017). Influences on the Test-Retest Reliability of Functional Connectivity MRI and its Relationship with Behavioral Utility. *Cerebral Cortex (New York, N.Y. : 1991)*, *27*(11), 5415–5429. http://doi.org/10.1093/cercor/bhx230

Poldrack, R. A., Congdon, E., Triplett, W., Gorgolewski, K. J., Karlsgodt, K. H., Mumford, J. A., et al. (2016). A phenome-wide examination of neural and cognitive function. *Scientific Data*, *3*, 160110. http://doi.org/10.1038/sdata.2016.110

Shen, X., Finn, E. S., Scheinost, D., Rosenberg, M. D., Chun, M. M., Papademetris, X., & Constable, R. T. (2017). Using connectome-based predictive modeling to predict individual behavior from brain connectivity. *Nature Publishing Group*, *12*(3), 506–518. http://doi.org/10.1038/nprot.2016.178

Shen, X., Tokoglu, F., Papademetris, X., & Constable, R. T. (2013). Groupwise whole-brain parcellation from resting-state fMRI data for network node identification. *NeuroImage*, *82*(C), 403–415. http://doi.org/10.1016/j.neuroimage.2013.05.081
